# The pharmacokinetics of mianserin suppositories for rectal administration in dogs and healthy volunteers: a pilot study

**DOI:** 10.1186/s40780-016-0046-7

**Published:** 2016-05-17

**Authors:** Shuichi Nawata, Noriko Kohyama, Naoki Uchida, Satoshi Numazawa, Masayuki Ohbayashi, Yasuna Kobayashi, Masanori Iwata, Takanori Nakajima, Hiroshi Saito, Akira Izuka, Toshinori Yamamoto

**Affiliations:** Pharmaceutical Department, Yokohama City University Medical Center, 4-57 Urafune, Minami-ku, Yokohama, 232-0024 Japan; Division of Clinical Pharmacy, Department of Pharmacotherapeutics, Showa University School of Pharmacy, 1-5-8 Hatanodai, Shinagawa-ku, Tokyo, 142-8555 Japan; Clinical Research Institute for Clinical Pharmacology and Therapeutics, Showa University Karasuyama Hospital, 6-11-11 Kitakarasuyama, Setagaya-ku, Tokyo, 157-8577 Japan; Division of Toxicology, Department of Pharmacology, Toxicology & Therapeutics, Showa University School of Pharmacy, 1-5-8 Hatanodai, Shinagawa-ku, Tokyo, 142-8555 Japan; Department of Clinical Pharmaceutics, Yokohama College of Pharmacy, 601 Matano-cho, Totsuka-ku, Yokohama, 245-0066 Japan; Clinical Pharmacy Educational Center, Nihon Pharmaceutical University, 10281 Komuro, Ina-cho Kita-adachi-gun, Saitama, 362-0806 Japan

**Keywords:** Delirium, Mianserin, Suppository, Pharmacokinetics

## Abstract

**Background:**

We formulated mianserin suppositories for the treatment of delirium and evaluated their pharmacokinetics by measuring plasma drug concentrations in dogs and healthy human volunteers.

**Methods:**

Mianserin suppositories were prepared by a melting technique using Tetramide® tablets and Witepsol H-15 as the suppository base. Pharmacokinetics of this 30-mg mianserin preparation were evaluated in three beagle dogs and three healthy adult males, in line with ethics committee approval. Plasma mianserin levels were determined using gas chromatography–mass spectrometry.

**Results:**

In dogs, the maximum plasma mianserin concentration (C_max_) was 1.3 ± 0.4 ng/mL, the time to C_max_ (t_max_) was 5.5 ± 4.3 h, and the area under the plasma concentration-time curve from 0 to 24 h (AUC_0-24_) was 18.9 ± 1.9 h・ng/mL. In humans, the C_max_ was 14.6 ± 6.3 ng/mL, the t_max_ was 8 h, and the AUC_0-24_ was 266 ± 103 h・ng/mL.

**Conclusions:**

The current study characterized the pharmacokinetics of mianserin suppositories in dogs and humans. As compared to oral administration, the suppositories produced a lower C_max_ and a delayed t_max_, although AUC_0-24_ values were comparable. It will be necessary to identify an appropriate dose that produces an adequate plasma mianserin concentration for effective and safe clinical use.

**Trial registration:**

UMIN000013853.

## Background

Typical antipsychotics such as haloperidol, atypical antipsychotics (risperidone, perospirone, quetiapine, and olanzapine), and tetracyclic antidepressants (mianserin and trazodone) have been used to treat delirium [1–3]. At present, haloperidol injection is the only treatment option available to patients who have difficulties with oral administration. Although haloperidol has been used widely to treat delirium, it can cause extrapyramidal disorder. Therefore, there is a need to develop alternative drugs for parenteral and/or non-tube administration in the general ward and at home.

Mianserin is a tetracyclic antidepressant with a weak affinity for cholinergic receptors; its antidepressant effect is considered to derive mainly from blockade of pre-synaptic alpha2-adrenoceptors, with a consequent increase in noradrenergic neurotransmission [4, 5]. There are several theories relating to the pathogenesis of delirium, including an alteration in serotonin neurotransmission [6]. The mechanism of mianserin-induced improvement of delirium remains unclear, but may be attributable to mianserin’s selective antagonism of 5-HT_2_ receptors [7].

Unlike other antipsychotics used to treat delirium, mianserin is unlikely to cause extrapyramidal effects. There is no high-quality clinical research evidence, such as a randomized controlled trial, for the efficacy of mianserin in the treatment of delirium. However, some non-randomized trials have reported the efficacy of mianserin for treating delirium. Sixty-two consecutive in-patients with a mean age of 80.7 years who were diagnosed with delirium were orally administered 10–90 mg mianserin/day (one administration in the evening) and assessed weekly using the Delirium Rating Scale over a 4-week study period. This study reported that mianserin was effective, particularly for the treatment of behavioral disturbances (85.5 % improvement rate) [8]. A separate open-label study demonstrated that 67 % of 46 patients (mean age: 63.0 years) who received 10–60 mg mianserin/day showed marked improvements [9]; these results were similar to those observed in a positive control group administered 2–6 mg haloperidol/day in which improvements were observed in 71 % of 17 patients.

We established a formulation for mianserin suppositories, prepared using a melting method and mianserin hydrochloride tablets: Tetramide® and Witepsol H-15 (Vosco®) [10]. H-15 was selected as the suppository base because the average drug release rate from mianserin-H15 suppositories is higher than that from mianserin-W35 or mianserin-S55 [10].

Rectal administration partially avoids the first pass effect, resulting in higher blood levels of drugs that are subject to extensive first pass metabolism following oral administration. A study in six healthy male subjects indicated a first-pass fraction of approximately 32 % following oral administration of mianserin hydrochloride [11]. Therefore, rectal administration of mianserin may result in a higher plasma drug concentration than that produced by oral administration, which may induce over-sedation. Therefore, prior to clinical application, we needed to characterize the pharmacokinetic profile of mianserin after rectal administration using a suppository.

The present study characterizes the pharmacokinetics of a mianserin suppository in an experimental animal prior to conducting a human study. Rats, rabbits, and dogs are frequently used for pharmacokinetic studies of suppositories. We selected dogs to test these human-sized suppositories. Secondly, we characterized the pharmacokinetics of mianserin after administering the rectal suppository in healthy adult volunteers. The results of this study provide information about the appropriate dose for future studies of the efficacy of mianserin suppositories in the treatment of delirium in patients who are unable to take oral medication.

## Methods

### Materials

Mianserin hydrochloride 10-mg tablets (Tetramide®) were purchased from MSD K.K. (Tokyo, Japan). Witepsol H-15 (Vosco® H-15), a hard fat suppository base, was purchased from Maruishi Pharmaceutical Co., Ltd. (Osaka, Japan). Mianserin hydrochloride standard was purchased from Sigma-Aldrich, Inc. (MO, USA). Mianserin-d3 dihydrochloride (1,2,3,4,10,14b-hexahydro-2-(methyl-d3)-dibenzo[c,f]pyrazino[1,2-a]azepine hydrochloride) was purchased as an internal standard from Santa Cruz Biotechnology, Inc. (Texas, USA). Methanol (high-performance liquid chromatography [HPLC] grade), n-hexane (HPLC grade), and isoamyl alcohol were purchased from WAKO (Osaka, Japan). All other chemicals were analytical grade. Pooled human plasma was purchased from Cosmo Bio Co., Ltd. (Tokyo, Japan).

### Suppository preparation

In this study, we prepared two types of suppositories in a clean room (class 10,000) at the Pharmaceutical Department, Yokohama City University Medical Center. Mianserin tablets were ground to a powder using a pestle and mortar and sifted using a 50-μm sieve. One gram of the mianserin tablet powder contained 100 mg mianserin hydrochloride. Witepsol H-15 base was melted at 40 °C and then the powder was added and mixed by stirring. Mianserin 30 mg suppositories were composed of a 1.1 g base and 0.3 g mianserin tablet powder. The molten mixture (1.4 mL) was aspirated into plastic suppository molds (1.35 mL; Kanae Co., Ltd., Osaka, Japan) using a glass injection syringe and allowed to solidify at room temperature. The suppositories were wrapped in aluminum foil to protect them from light and stored at 4 °C until use.

### Pharmacokinetic study in dogs

Six male beagle dogs aged 19–20 months were fasted from 6 p.m. on the day before mianserin administration to 8 h after administration. The dogs were randomized into two groups with three dogs in each group; one group received oral mianserin tablets and the other group received a rectal mianserin suppository.

Blood samples (5 mL) were collected before and 0.5, 1, 2, 4, 8, and 24 h after drug administration, transferred to tubes containing heparin sodium, inverted, and stored in an ice-cold box. Plasma was subsequently prepared and stored at −20 °C until it was analyzed.

The animal experimental procedures were conducted in accordance with the Regulations of the Ethics Review Committee for Animal Experimentation, and the Rules for the Approval of Animal Experimentation of KAC Co., Ltd. (Kyoto, Japan). The experimental protocol was approved by the animal experiment ethics committee of Showa University.

### Pharmacokinetic study in humans

This open-label, single-dose study was registered as a University Hospital Medical Information Network Clinical Trials Registry Clinical Trial (ID: UMIN000013853). The study was conducted according to the ethical principles of the Declaration of Helsinki and the Japanese Ministry of Health, Labour and Welfare. The study protocol was approved by the ethical committees of Showa University Karasuyama Hospital (receipt no. B-2013-021). Written informed consent was obtained from the participants, who were healthy male volunteers aged between 20 and 45 that were considered competent to provide consent, able to adhere to the rules of this study, and able to report their own symptoms; all subjects were judged eligible by the investigator after several medical check-ups. Subjects were excluded from study participation if their medical history was inappropriate (for example, a history of drug abuse, alcoholism, any kind of heart, liver, kidney, lung, eye, blood diseases or digestive system disorders, etc.) or if they ingested medicines during the study. Further exclusion criteria were as follows: smoker; any drug allergy; regular use of excessive alcohol with no abstention for the study period; participation in any clinical trial within the last 3 months; and subjects deemed inadequate for enrollment by the investigators. The target sample size was three. This was the minimum number of subjects and was chosen because this study was the first pharmacokinetic analysis of rectal mianserin administration. The subjects fasted for 14 h prior to drug administration until 3 h after administration. Suppositories (30 mg) were stored at 4 °C and brought to room temperature 30 min prior to administration to each subject by a nurse at 9 a.m. Blood samples (4 mL) were collected in heparinized tubes before and 0.5, 1, 2, 4, 8, and 24 h after mianserin administration. The samples were immediately centrifuged at 3,000 rpm for 10 min at 4 °C. Plasma was transferred to another two sample tubes and immediately frozen at −30 °C.

#### Outcomes

Primary outcomes were pharmacokinetic parameters (maximum plasma mianserin concentration [C_max_], time of C_max_ [t_max_], and area under the plasma concentration-time curve from 0 to 24 h [AUC_0-24_]). Secondary outcomes were self-assessment of sedation and safety profiles.

#### Self-assessment of sedation

Self-assessment of sedation was conducted prior to mianserin administration and 1, 2, 4, 8, and 24 h after administration; this was carried out 5 min before blood sampling to avoid altering the assessment. A visual analog scale, based on the Bond & Lader scale [12] translated into Japanese [13], was used to evaluate subjective sedation. The questionnaire included 16 question pairs. Between the question pair (for example, “alert” at the left-hand side and “drowsy” at the right-hand side), there was a 100 mm-long ungraded horizontal line. Each subject used a vertical line (|) to indicate their own estimate of sedation. The distance along the 100-mm line from the more sedate state to the subject’s mark was used for analysis. The average value from nine of 16 items was calculated as the sedation score at each time-point. The subjects were not told the items that would be measured or the direction of the measurement. New questionnaires were distributed to subjects at every time point so that they did not have access to their previous estimates.

#### Safety

Safety was evaluated using the following approaches: 1) medical interview and examination prior to administration and 4 and 24 h after administration; 2) vital signs (pulse and blood pressure in the sitting position after 5 min rest) and the axillary temperature before administration and at 4 and 24 h after administration; and 3) survey of subjective symptoms before administration and at 1, 2, 4, 8, and 24 h after administration.

### Plasma mianserin determination

The samples were transferred to the Division of Clinical Pharmacy, Department of Pharmacotherapeutics, Showa University School of Pharmacy, where the plasma mianserin concentration was determined using gas chromatography–mass spectrometry (GC-MS) [14].

Primary stock solutions of mianserin and mianserin-d3 dihydrochloride were prepared in methanol at a concentration of 1 mg/mL and further dilutions to the desired concentrations were made using methanol. These solutions were stored protected from light at approximately −20 °C.

After the internal standard (mianserin-d3; 50 μL of 0.2 ng/μL) was added to the 500-μL sample, it was mixed with 500 μL 2 M NaOH and vortexed [15]. The mixture was diluted with 5 mL hexane-isoamyl alcohol (99:1) and shaken for 30 min. After centrifuging the sample at 2000 *g* for 10 min, the upper layer was transferred to a new glass tube and evaporated under nitrogen at 40 °C. The evaporated extract was dissolved in 100 μL methanol.

A calibration curve was prepared using dog or human plasma (450 μL) containing mianserin (50 μL) to achieve concentrations of 15, 7.5, 3.75, 1.875, 0.938, 0.469, and 0.234 ng/mL. These values were plotted on the x-axis, while the y axis was used to plot the area ratio (area of the mianserin peak/area of the internal standard).

A GCMS-QP2010 Ultra system (Shimadzu, Kyoto, Japan) equipped with a Rtx-5MS column (30 m × 0.25 mmφ × 0.25 μm; Shimadzu GLC, Tokyo, Japan) was used. The initial column temperature was set at 90 °C for 1 min and then ramped at 50 °C/min to 180 °C, where it was held for 10 min; thereafter, the temperature was ramped again at 10 °C/min to 300 °C and held for 5 min. Helium with a constant flow of 1.52 mL/min and a pressure of 108.6 kPa was used as the carrier gas. A sample volume of 1 μL was injected in a splitless mode, with a 1-min sampling time. MS employed an ion source temperature of 230 °C and an electron voltage of 70 eV. Selective ion monitoring mode was used with m/z = 264 for mianserin and m/z = 267 for mianserin-d3.

Mianserin and internal standard peaks were not detected in a blank sample (plasma) or a zero concentration sample (blank sample with internal standard). At a signal/noise ratio = 3, the limit of detection was 0.2 ng/mL. The limit of quantitation was 0.234 ng/mL. The correlation coefficient of the calibration line was r = 0.999, and the mean accuracy was 7 %.

### Data analysis

The C_max_ and t_max_ were obtained by inspection of the plasma concentration-time curve. The trapezoidal method was used to calculate AUC_0-24_. The AUC from time zero to infinity (AUC_∞_) was calculated as the sum of AUC_0–24_ plus the ratio of the last measurable concentration to the elimination rate constant (ke). Because data from the elimination phase were insufficient to estimate ke, we used the reported ke value (0.039, calculated by dividing 0.693 by 18 h, which is half the time reported in the Tetramide® insert). According to the principles of pharmacokinetics, ke values do not depend on the route of administration.

To estimate the pharmacokinetic parameters of mianserin after administration by suppository, the 1-compartment model with first-order absorption equation (1) was fitted using a nonlinear least-squares regression analysis, which was conducted using the MULTI program [16]:

1 $$ \mathrm{C}\left(\mathrm{t}\right)=\mathrm{D}\cdotp \mathrm{k}\mathrm{a}\cdotp \mathrm{F}/\mathrm{V}\mathrm{d}/\left(\mathrm{ka}\hbox{-} \mathrm{k}\mathrm{e}\right)\times \left\{ \exp \left(\hbox{-} \mathrm{k}\mathrm{e}\cdotp \mathrm{t}\right)\hbox{-} \exp \left(\hbox{-} \mathrm{k}\mathrm{a}\cdotp \mathrm{t}\right)\right\} $$

where C represents the plasma mianserin levels, t represents time, D represents the dose, ka is the absorption rate constant, F is the bioavailability, Vd is the volume of distribution, and ke is the elimination rate constant. For these calculations, we fixed ke according to the reported value (0.039).

We used the obtained parameters and equation (1) to simulate the time-dependent changes in mianserin levels following mianserin suppository administration. The target plasma mianserin levels were 20–40 ng/mL and 60–80 ng/mL. The former is the steady state level achieved by daily oral administration of 30 mg Tetramide® tablets to healthy adults. The latter was set in reference to previous studies of plasma mianserin levels 11–13 h after once-a-day oral mianserin administration and the effect of mianserin on delirium [9, 17].

## Results

### Dog pharmacokinetics

**Fig. 1:**
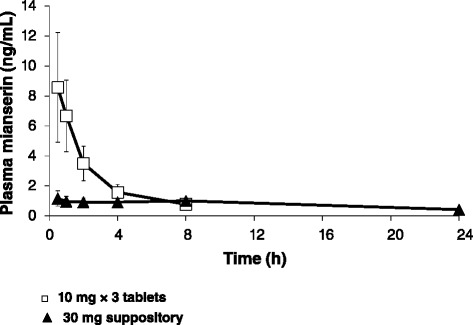
Pharmacokinetics of 30 mg mianserin after rectal and oral administration to beagle dogs. Data show the mean ± SD, *n* = 3

The plasma concentration-time profiles after administration of 30 mg mianserin by rectal suppository and oral tablet are shown in Fig. 1. After oral administration, the plasma mianserin level reached C_max_ at 30 min and then rapidly decreased. By 24 h after oral administration, the plasma mianserin level was below the lower limitation of quantitation. Plasma concentrations after rectal administration were lower than those observed after oral administration and these remained at 1 ng/mL for 8 h. By 24 h after rectal administration, the plasma mianserin concentration was 0.42 ± 0.07 ng/mL, higher than that observed after oral administration. The dog pharmacokinetic parameters are summarized in Table 1. Rectal administration resulted in a delayed t_max_, a lower C_max_, and an AUC_0–24_ value that was 70 % of that observed following oral administration.

**Table 1:** Pharmacokinetics of mianserin after a single administration to beagle dogs

	t_max_ (h)	C_max_ (ng/mL)	AUC_0-24_ (h • ng/mL)	AUC_∞_ (h • ng/mL)
Suppository (30 mg)	5.5 ± 4.3	1.3 ± 0.4	18.9 ± 1.9	29.5 ± 3.3
Oral tablets (3 × 10 mg)	0.5	8.6 ± 3.7	27.0 ± 8.6	23.8 ± 7.7

Mean ± SD, *n* = 3

### Human pharmacokinetics

**Fig. 2:**
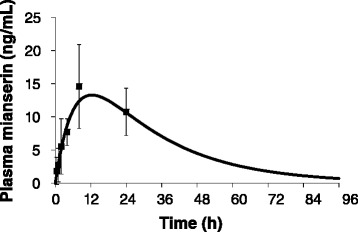
Pharmacokinetics of 30 mg mianserin after rectal administration in healthy adult males. Closed squares represent the data from healthy adult males receiving 30 mg mianserin by rectal administration. Data show the mean ± SD, *n* = 3. The solid line represents the curve fitted according to the 1-compartment model with first-order absorption equation (1). In order to estimate ka and Vd, mianserin pharmacokinetic data after rectal administration were fitted to equation (1) by nonlinear least-squares regression analysis with a fixed ke value. The estimated values of ka and Vd/F were 0.146 ± 0.052 1/h (mean ± SD, *n* = 3) and 1395 ± 497 L, respectively

Three healthy male Japanese subjects with a mean age of 24.7 years (range: 23–26 years), a mean height (± standard deviation [SD]) of 177.3 ± 5.9 cm, and a mean weight of 72.1 ± 7.1 kg completed this study. Their mean (± SD) plasma mianserin concentration-time profiles after administration of a rectal suppository are shown in Fig. 2, indicating that absorption of mianserin commenced immediately after rectal administration of the suppository and then slowly increased over time. Consistent with the dog data, the t_max_ was delayed, the C_max_ was lower, and the AUC_0–24_ was 79 % of that estimated from the available human oral data (Table 2). The estimated AUC_∞_ value after administration of a rectal suppository was 124 % of the AUC_∞_ value reported in the package insert for oral administration (Table 2).

**Table 2:** Pharmacokinetics of mianserin after a single administration to healthy male subjects

	t_max_ (h)	C_max_ (ng/mL)	AUC_0-24_ (h • ng/mL)	AUC_∞_ (h • ng/mL)
Suppository (30 mg)	8^a)^	14.6 ± 6.3^a)^	266 ± 103^a)^	541.8 ± 189.4^a)^
Oral tablets (30 mg)	2.0 ± 0.1^b)^	40.7 ± 2.6^b)^	338.5^c)^	435.4 ± 35.3^b)^

a) The present study data, expressed as mean ± SD, *n* = 3

b) Data derived from the package insert of Tetramide® Tablets

c) Values estimated from the plasma concentration profile in the Tetramide® Tablet package insert

**Fig. 3:**
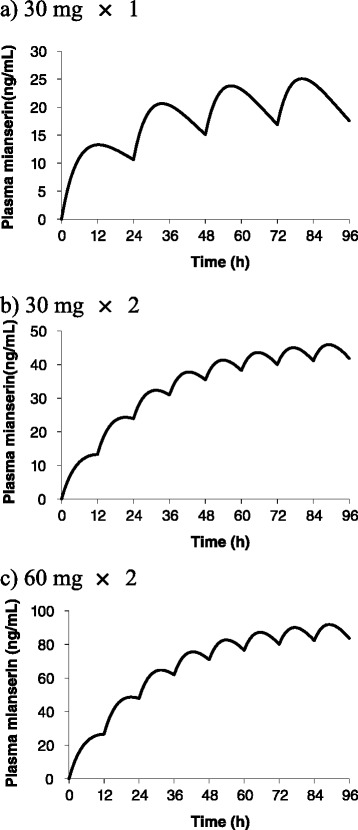
Predicted pharmacokinetics after rectal administration of mianserin by suppository. **a** 30 mg × 1, **b** 30 mg × 2, **c** 60 mg × 2

The estimated values of ka and Vd/F were 0.146 ± 0.052 1/h (mean ± SD, *n* = 3) and 1395 ± 497 L, respectively. The fitted curve and observed mianserin levels are shown in Fig. 2. Using the reported ke value and parameters estimated in this study, we simulated the mianserin time profiles after daily administration for 4 days (Fig. 3). The schedule of 30 mg daily achieved a level of 17–25 ng/mL. A twice-daily dosage of 30 mg was necessary to obtain plasma mianserin levels of 40–45 ng/mL. A twice-daily dosage of 60 mg was predicted to result in plasma mianserin levels of 80–90 ng/mL.

Sedation scores (mean ± SD, *n* = 3) at 30 min, 1, 2, 4, 8, and 24 h after drug administration were 53.7 ± 3.8 mm, 56.7 ± 7.4 mm, 57.3 ± 7.0 mm, 59 ± 6.6 mm, 55 ± 5.6 mm, and 48.7 ± 4.2 mm, respectively; these did not differ significantly from those recorded prior to drug administration (51 ± 3.6 mm; repeated-measures ANOVA).

One of the study subjects reported adverse events associated with administration of mianserin. He complained of an unpleasant mood, followed by a transiently disturbed consciousness during lunch (4 h after administration) and supper (8 h after administration). His pulse and blood pressure decreased and he recovered after bed rest. A medical examination 24 h after administration found that all of his vital signs were normal and he did not report any subjective symptoms.

## Discussion

To characterize mianserin suppository pharmacokinetics, we conducted this pilot study of a single rectal administration of a mianserin (30 mg) suppository in dogs and healthy adult volunteers. The study showed that blood mianserin concentrations increased more slowly following rectal administration than after oral administration.

This is the first study, to our knowledge, to provide pharmacokinetic data relating to mianserin in dogs. The values of t_max_, C_max_, and AUC_0-24_ following oral administration to dogs were 25, 21, and 8 % of the corresponding human values, respectively (Tables 1 and 2).

The dose of mianserin employed in our subsequent human study was determined by extrapolating the ratio of rectal to oral exposure observed in the dog study and considering this in the context of previous oral studies in humans. The efficacy of 10–90 mg mianserin/day had been previously reported in the context of delirium. An open-label clinical trial on aged patients with delirium demonstrated that 49/62 patients (85.5 %) were judged responders; doses of ≤ 30 mg mianserin were also effective in 85.7 % of these responders [8]. A separate open-label study on aged patients showed an effect of mianserin on delirium [7]. After an initial dose and titration period, administration of 25–27 mg mianserin/day improved delirium symptoms in 24/26 (92 %) of these patients. Nakamura et al. also reported that an average dose of 25–27 mg mianserin/day was effective in 85 % of 46 cases [9]. Therefore, we selected a rectal mianserin dose of 30 mg for this study.

In humans, the plasma mianserin concentration also increased slowly after rectal administration and while the mean t_max_ was markedly delayed, the AUC_0-24_ was 80 % of that observed after oral administration. The dog and human pharmacokinetic profiles of mianserin suppositories were therefore similar; both showed slower and more sustained drug release, with a markedly delayed t_max_, a higher concentration 24 h after administration, and comparable AUC_0-24_ values, as compared with oral mianserin.

The sedative effect of mianserin, which reflects its antagonist activities at histamine H_1_ receptors and alpha1-adrenoceptors, was subjectively evaluated as a secondary outcome following administration of mianserin suppositories. In general, the lower C_max_ observed following rectal (as compared to oral) administration and the small number of subjects meant that we did not observe significant sedative effects in the present study. In one subject, sedation scores were higher 30 min after administration than at baseline, with drowsiness reaching a maximum value (100 mm) 2 h after administration. This subject had a mianserin level that was three-fold higher than that of the other two subjects at this time-point; this is likely to have contributed to this sedative effect. Furthermore, this subject fainted 4 h and 8 h after administration when his mianserin levels were 10 ng/mL and 21.9 ng/mL, respectively; these levels were two-fold higher than those of the other subjects and approached 50 % of the C_max_ reported after oral administration of 30 mg mianserin (45.2 ng/mL; Tetramide® insert). No rapid increase in the plasma mianserin level was observed, suggesting that this did not cause the adverse events. Nevertheless, attention needs to be paid to side effects in order to ensure the safe use of mianserin suppositories.

We found that plasma concentrations of mianserin 24 h after rectal administration were still high, and the mianserin suppository AUC_∞_ was comparable to that observed following oral administration, despite the lower C_max_. Therefore, it is possible that the plasma level at steady-state will be much higher than that after a single administration. Furthermore, it is possible that a higher dose or multiple dosing will be required in order to achieve a sufficient plasma level to treat delirium. For reference, we conducted an estimation of pharmacokinetic parameters during multiple-dosing using a 1-compartment model with first-order absorption equation. Once-daily administration of 30-mg suppositories achieved a plasma concentration of 20 ng/mL, which corresponded to the level produced by oral dosing in healthy young adults (Tetramide® insert) within 72 h of administration. Twice-daily administration of 60 mg using suppositories achieved a plasma concentration of 60–80 ng/mL, which corresponded to the level observed after oral dosing of patients with delirium [9, 17] within 36 h of administration. Further studies are needed to characterize the rectal dose-plasma concentration relationship, plasma concentrations beyond 24 h after administration, and steady-state pharmacokinetics after multiple doses.

## Conclusions

In conclusion, the results of the current study indicate that rectal administration of a suppository prepared using mianserin hydrochloride tablets and Witepsol H-15 results in slow systemic mianserin exposure and a lower C_max_ as compared with oral tablet administration. Our findings provide a preliminary indication that rectal suppository administration could provide a comparable exposure to mianserin and might offer an alternative route in patients in which oral administration is not possible. Future studies should aim to clarify the relationship between the rectal mianserin dose and the blood concentration in order to use this route effectively and safely.

## Abbreviations

AUC_0-24_: Area under the concentration-time curve from time zero to 24 h
AUC_∞_: Area under the concentration-time curve from time zero to infinity
C_max_: Maximum plasma concentration
GC-MS: Gas chromatography–mass spectrometry
t_max_: Time to reach the maximum plasma concentration
